# Role of high-resolution ultrasound in detection and monitoring of peripheral nerve tumor burden in neurofibromatosis in children

**DOI:** 10.1007/s00381-020-04718-z

**Published:** 2020-06-19

**Authors:** Natalie Winter, Maike F. Dohrn, Julia Wittlinger, Alexander Loizides, Hannes Gruber, Alexander Grimm

**Affiliations:** 1 Department of Neurology and Hertie Institute for Clinical Brain Research (HIH), University of Tübingen, Hoppe-Seyler-Str. 3, 72076 Tübingen, Germany; 2 University Hospital of the RWTH Aachen University, Aachen, Germany; 3 Department of Radiology, University of Innsbruck, Innsbruck, Austria

**Keywords:** High resolution nerve ultrasound, Neurofibroma, Schwannoma, Neurofibromatosis

## Abstract

**Purpose:**

Peripheral nerve sheath tumors are hallmark findings in neurofibromatosis types 1 and 2. With increasing size, they typically lead to neurological symptoms, and NF1 patients have a lifetime risk of 8–13% for developing malignant peripheral nerve sheath tumors. Medical imaging is therefore highly needed for early detection and exact localization of symptomatic or potentially malignant tumors. This review will give an overview of the ultrasound characteristics of peripheral nerve sheath tumors and findings in patients with neurofibromatosis types 1 and 2.

**Methods:**

A systematic search of electronic databases, reference lists, and unpublished literature was conducted including the keywords “schwannoma,” “neurofibroma,” “neurofibromatosis,” “benign and malignant peripheral nerve sheath tumor.”

**Results:**

The high-resolution allows a clear analysis of tumor echotexture, definition of margins, and the relation to the parent nerve. The use of color duplex/Doppler and contrast agent adds valuable information for the differentiation of benign and malignant tumors.

**Conclusion:**

High-resolution ultrasound is a well-established, non-invasive, and easily repeatable first-line tool in diagnostic procedures of soft tissue tumors.

## Introduction

Neurofibromatosis (NF) types 1 and 2 belong to a heterogeneous group of hereditary phacomatosis syndromes that lead to tumor formation in both the central and peripheral nervous systems with an autosomal dominant mode of inheritance. Both types are rare diseases with different prevalence (NF1 1, 2500–3000; NF2 1, 35,000) and sporadic occurrence in up to 50% [18, 19, 21, 23].

With regard to manifestations of the peripheral nervous system, the defining feature of neurofibromatosis type 1 is peripheral nerve sheath tumors, typically neurofibromas and malignant peripheral nerve sheath tumors (MPNST) [11]. MPNST develop in 1–2% of NF1 patients [5, 12], often arising from plexiform neurofibromas [2]. The clinical presentation and ultrasound findings in NF1 have a wide variability. While several clinical signs of NF1 are already present at birth, others develop over time. In particular, neurofibromas occur and grow with aging [11, 21].

NF2 patients more frequently develop schwannomas and have a lifetime risk of up to 66% for axonal polyneuropathy [1, 2].

The primary aims of imaging-based diagnostics in patients with neurofibromatosis are (1) the early identification of potential tumor transformation signs into a malignant peripheral nerve sheath tumor, which is most important in NF1 patients; (2) the exact determination of localization, size, and extent of benign and malignant nerve tumors for visualization of tumor-related complications and therapy planning (i.e., surgery); and (3) the reliable detection of soft tissue tumor and quantification of tumor load as a basis for further therapy or genotype-phenotype studies [21].

Whole-body MRI is frequently used for staging and follow-up [2], but with the need for anesthesia, it is associated with a high procedural burden especially in younger children.

High-resolution ultrasound (HRUS) is a well-established method for the examination of peripheral nerves. Several studies have demonstrated its high value for diagnosis and therapy planning in nerve trauma, polyneuropathies, mononeuritis, and nerve tumors [2, 7, 17, 30]. Almost all peripheral nerves and the brachial plexus can be visualized throughout their entire course. The nerve is evaluated in the short- and long axes and the cross-sectional area (CSA) of the nerve and—if necessary—of single fascicles can be measured. Normal values for CSA of peripheral nerves and fascicles are well-established by several groups [4, 8, 9], and recently, normal values in children for different ages have been published [22].

Due to a constant improvement of ultrasound devices, an axial resolution of up to 30 μm is now possible [24, 27]. The use of color Doppler and duplex mode adds useful information on vascularization of nerves and especially of nerve tumors. Further new methods were established, i.e., contrast-enhanced ultrasonography and elastography. The method is cost-effective, easily accessible, and radiation-free and thus also suited for the examination of children. The main obstacle in any ultrasound-based examination is its limited penetration depth, making it less accurate whenever deeper structures are of interest, e.g. tumors in the paravertebral region, the lumbar plexus, or thorax.

## General aspects—ultrasound of peripheral nerve sheath tumors

The most common benign peripheral nerve sheath tumors are neurofibromas and schwannomas, also called neurinoma or neurolemoma. The differentiation of these two tumor types can be challenging, but several ultrasound features are more common in either of them (Table 1).

**Table 1:** Typical ultrasound characteristics of benign and malignant peripheral nerve sheath tumor

	Benign	Malignant (MPNST)
Tumor size	< 5 cm	> 5 cm (> 3.3 cm)
Growth rate	Slowly growing, years	Fast-growing within weeks to months
Margins	Well-defined	Irregular, ill-defined
Peritumoral edema	Not present	Present
Echogenicity and echotexture	Homogenous “ancient” schwannoma: hyperechoic areas, cysts, calcification	Inhomogeneous, calcification, cysts, central necrosis, hemorrhage
Localization in relation to the nerve	Schwannoma: more eccentric, displacement of fascicles Neurofibroma: concentric, “interfere” with fascicles	Infiltrative
Vascularization	Hierarchic, good vascularization (schwannomas), poor vascularization (neurofibromas)	Stenosis, occlusion, trifurcation, archaic vascular pattern
CEUS	No enhancement, homogenous enhancement (more common in schwannomas)	Peripherally enhancing + non-enhancing central area or diffusely enhancing mass + scattered non-enhancing areas and/or enhancement bridges

*CEUS*, contrast-enhanced ultrasonography; *MPNST*, malignant peripheral nerve sheath tumor

Both tumor types can occur sporadically, typically in young adults in the second and third decades of life [14]. These tumors grow very slowly, so that symptoms are seldom or can be totally absent. Irradiating pain provoked by pressure at the lesion site is the most frequent early symptom [17]. By increasing size, more fascicles are damaged by displacement or infiltration. Muscle atrophy and weakness, paresthesia, and hypesthesia can occur more typically in the advanced course of neurofibromatosis.

### Schwannoma

**Fig. 1:**
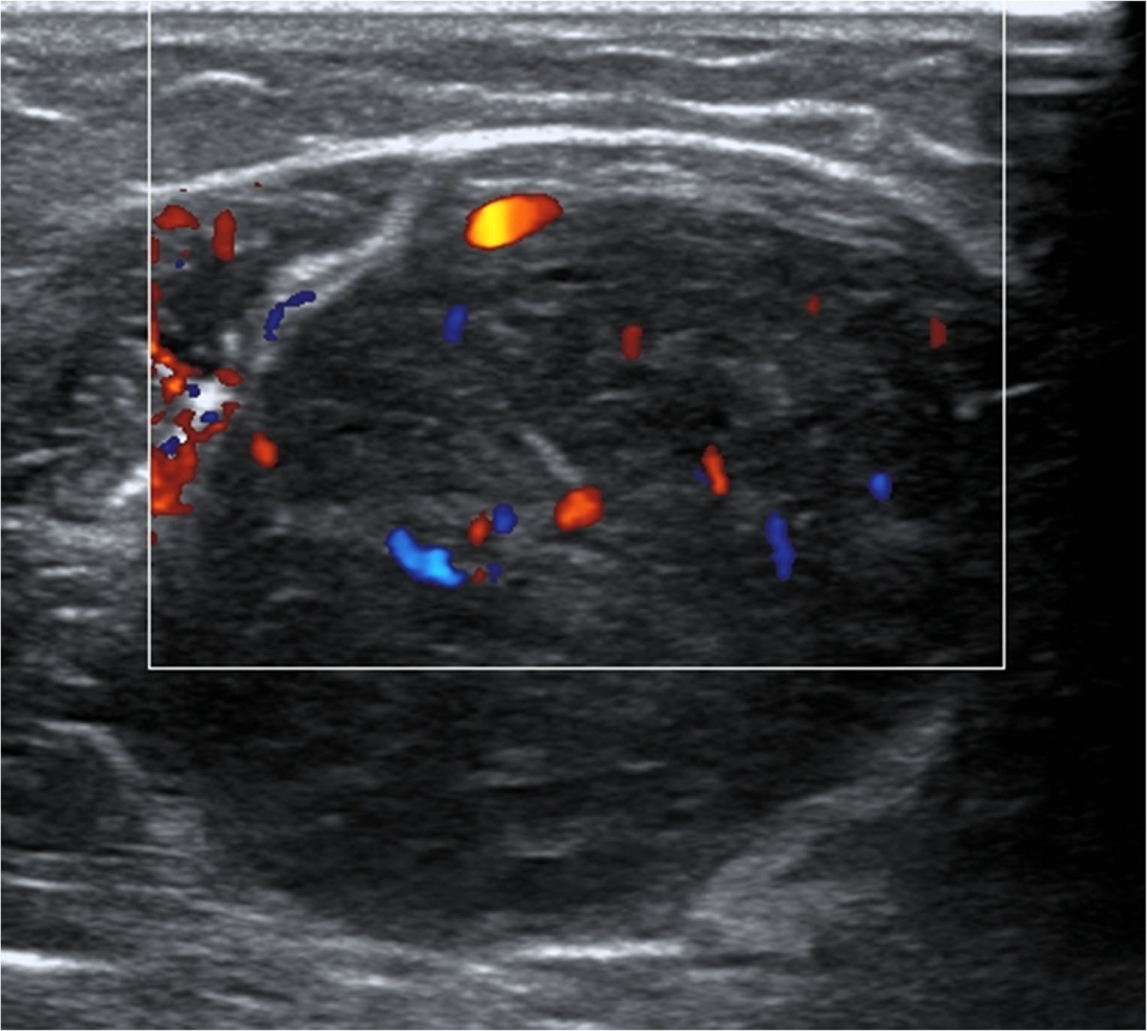
Color-mode image of a schwannoma. The ovoid, mostly hypoechoic tumor with its clear borders, arising from one fascicle, is the hallmark finding in peripheral NF2 nerves. However, a schwannoma can also be seen in other patients and occur as single lesion. Large tumors are mostly traversed by many vessels with regular blood flow

**Fig. 2:**
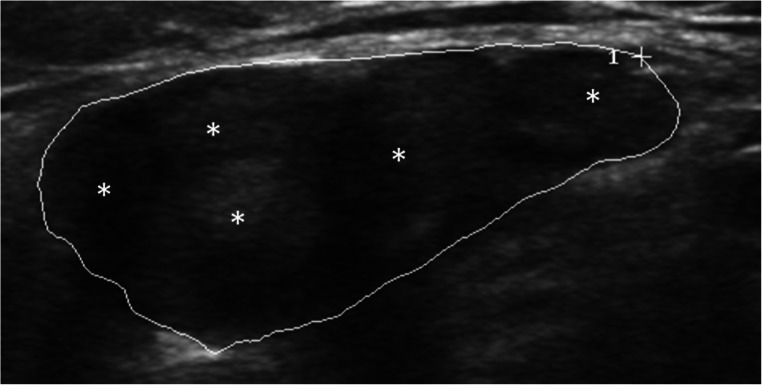
Neurofibromas are more heterogeneous than schwannomas with often mixed echointensity. Their borders are sharply delimited, but the lump-like shape is not as well-defined as in schwannomas. In contrast to schwannomas, several fascicles are involved (asterisks)

Schwannomas are typically solid tumors, which are characterized by a hypoechoic ovoid shape [10]. The margins are well-defined and, in most lesions, a posterior acoustic enhancement occurs [17]. The peripheral nerve entering and exiting the tumor can be detected in most cases. Eccentric tumor localization is typical in larger schwannomas and uncommon in neurofibromas [17, 20]. Schwannomas emerge from one fascicle, whereas solid neurofibromas often originate from a group of fascicles, which is represented by an onion-like texture with hyperechoic and hypoechoic tumor masses (Figs. 1 and 2) [17].

Timely progressed, namely “ancient,” and larger schwannomas can exhibit degenerative changes, such as internal bleeding, hypoechoic cavities, rarely calcifications, and hyperechoic areas [14, 17, 20], which can hamper the differentiation from malignant tumors. In larger schwannomas, an increased internal vascularization is common. Tumor vessels entering from the proximal and distal pole, monomorphic duplex waveform, and a hierarchic vasculature architecture are typical features of schwannomas [3, 10]. A target sign is normally found in neurofibromas but can rarely be present in schwannomas as well [18].

### Neurofibroma

Neurofibromas represent around 5% of benign soft tissue neoplasms [14]. Most lesions are solitary, mainly originate from small nerve branches, and are located subcutaneously. Besides dermal neurofibromas, diffuse and plexiform variants occur [10]. Like schwannomas, the solitary neurofibromas present as homogeneous, sharply delimited lesions [10, 14, 17]. In contrast to schwannomas, they grow concentrically in relation to the nerve [10, 20] and do no displace fascicles but “interfere” with them [10, 20]. A target sign, a hypoechoic peripheral zone, and a hyperechoic central zone are characteristics in neurofibromas [10, 14, 17].

In plexiform neurofibromas, HRUS mainly features serpentine-like, partly oval-shaped in length, mostly hypoechoic, well-confined tumors that arise from multiple fascicles [29]. Plexiform neurofibromas are pathognomonic for NF1 and have an increased risk for malignant transformation [5, 10, 14].

### Malignant peripheral nerve sheath tumor

**Fig. 3:**
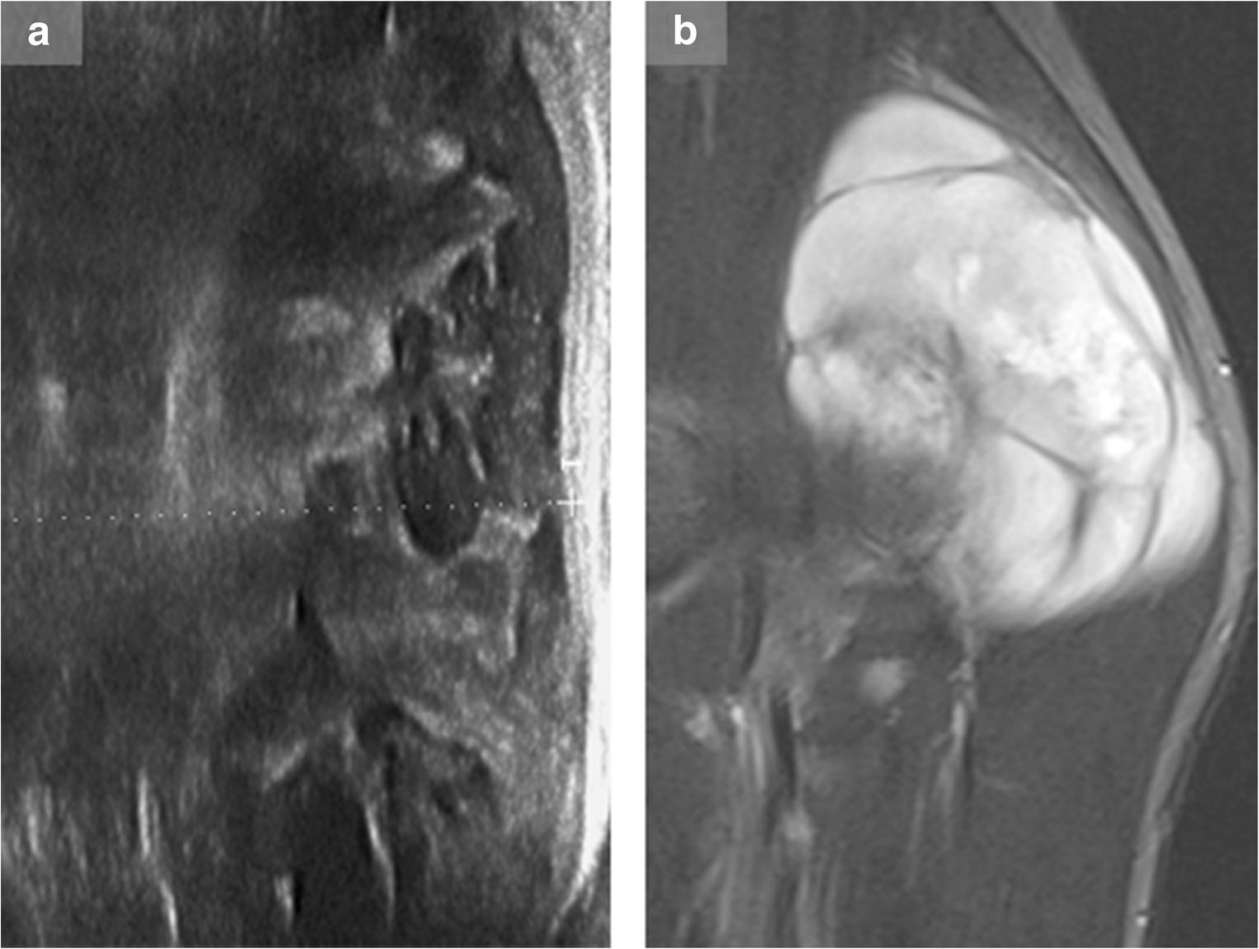
Tremendous growth of a lump-like mass, infiltrative aspects, changed vascularity, and random heterogeneity must raise attention to malignancy. In this figure, ultrasound (**a**) and corresponding MRI images (**b**; short TI inversion recovery (STIR)–weighted sequence of the same tumor, sagittal plane) of a malignant peripheral nerve sheath tumor in NF1 are shown

MPNST are characterized by fast and typically massive growth and an early incidence of neurological symptoms and pain [17]. Their appearances are very diverse and range from well-circumscribed to grossly infiltrating [18]. Heterogeneity with central necrosis, hemorrhages, and calcification on cross-sectional imaging is common in malignant lesions (Fig. 3). Similarly, calcification, more commonly associated with malignant lesions, can also be present in ancient schwannomas [10, 14, 21, 28]. An analysis of vascularization of musculoskeletal tumors revealed four major vessel characteristics (stenosis, occlusion, trifurcation, archaic vascular pattern), which proved helpful in differentiating benign from malignant lesions. The combination of any of these major characteristics showed the best results (sensitivity 94%, specificity 93%) [3], but further studies for peripheral nerve sheath tumors are needed.

Contrast-enhanced ultrasonography (CEUS) seems to add valuable information on the distinction between benign and malignant musculoskeletal tumors. Several criteria are considered for evaluation: the absence of contrast uptake had a sensitivity of 60% and a specificity of 68% for the diagnosis of a benign tumor. The area under the curve of contrast uptake and the slope and peak intensity were significantly higher in malignant in comparison with those in benign musculoskeletal tumors [6]. Another parameter, the perfusion pattern, was analyzed by Loizides and colleagues. The best-combined sensitivity (89%) and specificity (85%) was achieved by the combination of three features: size > 3.3 cm, mass location below the superficial fascia, and a perfusion pattern of either peripherally enhancing mass with non-enhancing central area or diffusely enhancing mass with scattered non-enhancing areas and/or enhancement bridges [15].

## Nerve ultrasound in neurofibromatosis 1

The most common peripheral nerve sheath tumors are neurofibromas in NF1. Plexiform neurofibromas are a pathognomonic finding occurring in 30 to up to 90% of patients [18, 19, 25, 26, 29]—depending on the reported study population. Schwannomas are seldomly found.

In the setting of neurofibromatosis, neurofibromas tend to be larger and have a higher incidence of malignant transformation [14]. Dermal neurofibromas occur in most adults with neurofibromatosis type 1, and cosmetic concerns as well as itching and stinging experiences contribute to a reduced quality of life [11].

**Fig. 4:**
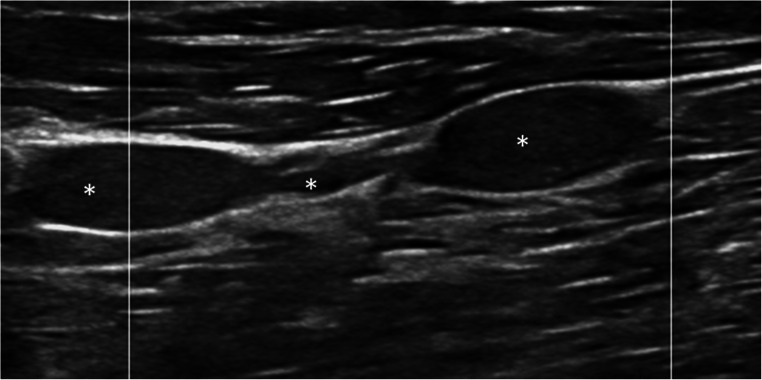
Plexiform neurofibroma in a patient with NF1 in the long axis. The serpentine-like neurofibromas are marked with asterisks

So far, only few ultrasound studies exist including patients with neurofibromatosis. HRUS findings vary among the patients and range from subtle nerve changes to moderately enlarged, but strictly localized neurofibromas, plexiform neurofibromas (localized or generalized), and large tumor masses with extensive growth [25, 26, 29] (Fig. 4). HRUS shows clear abnormalities in up to 94% of the patients, independent from the presence of symptoms [25].

Localized tumors outside the large nerve branches have been found in 60 to 90% of the patients examined by HRUS [25, 29]. In a study with asymptomatic patients, 35% had nerve enlargement without an abnormal fascicular pattern [25]. Due to the limited sample sizes, these data have to be proven in further multicenter studies.

With a lifetime risk of 8–13%, patients with neurofibromatosis show a high tendency of developing MPNST. The life span in these patients is reduced by 8 to 21 years due to a higher risk of developing a malignant tumor, i.e., breast cancer or MPNST [11, 12]. Conflicting reports about the prognosis and survival of NF1 patients with MPNST exist. A meta-analysis including 48 studies and more than 1800 patients in a time span of 50 years revealed a significantly higher odds ratio for overall survival (OR_OS_) and disease-specific survival (OR_DSS_) in the non-NF1 group (OR_OS_ = 1.75, 95% confidence interval = 1.28–2.39, OR_DSS_ = 1.68, 95% CI = 1.18–2.40). But in studies published in the last years, the survival in both patient groups has been converging due to an improved prognosis in NF1 patients [13]. Accurate screening and tight follow-up examinations are mandatory in these patients.

## Nerve ultrasound in neurofibromatosis 2

In patients with neurofibromatosis type 2, the main ultrasound finding is focal schwannoma within the nerve, whereas localized tumors outside the main branches are rarer than in NF1 (up to 40%) [29]. The nerve tumors arise from single fascicles and are hypoechoic and homogeneous, oval-shaped in length, with well-defined margins and normally strong vascularization [10]. Between the tumors, the studied nerves have a normal morphology and CSA. Malignant transformation has not been reported in NF2 [23]. Patients can develop a polyneuropathy in up to 66%, which seems to correlate with the cumulative burden of non-compressive fascicular microlesions (< 2 mm) within a nerve [2].

## Discussion

In neurofibromatosis, the nerve ultrasound can easily visualize significant mass growth non-invasively, especially in children. It is also capable to monitor stable disease and to detect subclinical nerve involvement and thus might avoid overwhelming MRI acquisition. The detection of distinct sonomorphological pattern (localized NF, plexiform NF, tumor load and mass, overall enlarged nerves) might play a role as a prognostic marker concerning MPNST development [25, 26, 29], but further studies are needed. From a future perspective, nerve ultrasound might find use as an intraoperative tool to determine the amount of tumor mass and its borders enabling a more precise resection [16]. However, if nerve growth is revealed by ultrasound, this tool might not be sensitive enough to distinguish between a benign mass increase or malignant transformation in all cases. Although the findings of infiltrative masses, archaic vessels, and overall heterogeneity point towards malignancy, PET CT/MR imaging and histological analysis are still highly recommended in such cases. The growing role of contrast-enhanced ultrasound in these cases is not yet clear and must be examined in future studies [10]. In summary, high-resolution nerve ultrasound is useful not only as a screening tool but also as an essential player in triaging and monitoring NF patients.

## Conclusion

HRUS is a very useful first-line tool for triaging and monitoring neurofibromatosis-associated peripheral nerve sheath tumors. In combination with other imaging modalities (MR, PET MR/CT), an early suspicion of malignant peripheral nerve sheath tumors is enabled, and further diagnostic and therapeutic procedures can be planned at rather early stages.
